# Researcher’s Perspective on Musculoskeletal Conditions in Primary Care Physiotherapy Units through the International Classification of Functioning, Disability, and Health (ICF): A Scoping Review

**DOI:** 10.3390/biomedicines11020290

**Published:** 2023-01-20

**Authors:** Héctor Hernández-Lázaro, María Teresa Mingo-Gómez, Sandra Jiménez-del-Barrio, Silvia Lahuerta-Martín, Ignacio Hernando-Garijo, Ricardo Medrano-de-la-Fuente, Luis Ceballos-Laita

**Affiliations:** 1 Faculty of Health Sciences, University of Valladolid, 42004 Soria, Spain; 2 Clinical Research in Health Sciences Group, University of Valladolid, 42004 Soria, Spain; 3 Ólvega Primary Care Health Center (Soria, Spain), Soria Health Care Management, Castilla y León Regional Health Management (SACYL), 47007 Valladolid, Spain

**Keywords:** ICF, musculoskeletal conditions, primary care, physiotherapy, outcome measures

## Abstract

(1) Background: Musculoskeletal disorders are the second cause of disability in the world. The International Classification of Functioning Disability and Health (ICF) is a tool for systematically describing functioning. Outcome measures for musculoskeletal disorders and functioning concepts embedded in them have not been described under the ICF paradigm. The objective of this scoping review was to identify ICF categories representing the researcher’s perspective and to compare them with the ICF core set for post-acute musculoskeletal conditions. (2) Methods: This review was conducted as follows: (a) literature search using MEDLINE/PubMed, CINAHL, Web of Science, and Scopus databases; (b) study selection applying inclusion criteria (PICOS): musculoskeletal conditions in primary care, application of physiotherapy as a treatment, outcome measures related to functioning, and experimental or observational studies conducted in Western countries during the last 10 years; (c) extraction of relevant concepts; (d) linkage to the ICF; (e) frequency analysis; and (f) comparison with the ICF core set. (3) Results: From 540 studies identified, a total of 51 were included, and 108 outcome measures were extracted. In the ICF linking process, 147 ICF categories were identified. Analysis of data showed that 84.2% of the categories in the ICF core set for post-acute musculoskeletal conditions can be covered by the outcome measures analyzed. Sixty-eight relevant additional ICF categories were identified. (4) Conclusion: Outcome measures analyzed partially represent the ICF core set taken as a reference. The identification of additional categories calls into question the applicability of this core set in primary care physiotherapy units.

## 1. Introduction

Musculoskeletal disorders are a wide range of conditions that affect an estimated 1.7 billion people and are considered the second leading cause of disability worldwide [1]. This type of disease causes pain and physical deficits that limit the functional capacity of patients, impacting their social context and affecting their personal life. Furthermore, musculoskeletal pathology is also one of the main causes of chronic pain and contributes to the perpetuation of this clinical entity [2,3].

The high prevalence of these disorders constitutes one of the main reasons for assistance in primary care health services, reporting 18% of all general consultations [4]. Mainly the physiotherapy service is in charge of managing these alterations through conservative treatment and health education. The physiotherapeutic approach to musculoskeletal problems not only focuses on the functional status of the patient, but also takes into account a variety of contributors such as biomedical, psychological, or social factors [5].

The International Classification of Functioning (ICF) was proposed by the World Health Organization (WHO) in 2001 as a reference system for functioning. ICF combines categories and qualifiers to describe functioning and disability and relates these concepts to the patient’s context. In this way, ICF categories are structured with the following components: body structures and functions, activities and participation, environmental factors, and personal factors. Qualifiers provide a measure of the severity [6].

Since its approval, the clinical use of the ICF has been expanding, especially in rehabilitation and outcome assessment. However, their level of implementation is very heterogeneous when comparing countries, with Sweden and Australia reporting the most widespread use in clinical settings [7]. The development of ICF core sets promoted by the WHO and the ICF Research Branch has enhanced the likelihood of ICF use in multiple clinical settings [8]. Two ICF core sets were already developed for musculoskeletal conditions, targeting acute and post-acute stages [9,10,11]. However, there is a lack of an ICF-based tool for these disorders directly applicable at the community level. It is also not known whether the assessment instruments frequently used in this clinical setting cover the essential aspects of functioning in patients with musculoskeletal problems. In a recent study involving primary care physiotherapists, it was shown that current ICF core sets for musculoskeletal conditions only partially represented the perspective of these professionals, so the need to develop a tailored ICF core set for this clinical context was raised [12].

According to the methodology proposed by Selb et al., [13] preliminary studies for the development of ICF core sets aim to capture the perspectives of researchers, professionals, patients, and clinical settings. To describe the researcher’s perspective, a scoping review of outcome measures in the scientific literature is needed. It is assumed that researchers consider the functioning-related measures they use to be relevant.

The objective of this study was to describe the researcher’s perspective on the management of musculoskeletal conditions in a primary care physiotherapy clinical setting in terms of ICF. Specific objectives were:

1. To identify the most frequent functioning concepts embedded in outcomes measures used when studying the target clinical context;

2. To link functioning concepts to ICF and compare them with the ICF core set for post-acute musculoskeletal conditions;

3. To assess the ability of the identified outcome measures to cover functioning aspects included in the ICF core set taken as a reference; and

4. To contribute to the development of a tailored ICF core set for primary care physiotherapy units by identifying additional ICF categories from outcome measures.

## 2. Materials and Methods

### 2.1. Study Design

This review was conducted following the methodology described by the ICF Research Branch [13] and was composed of five parts: (1) literature search study selection, (2) extraction of relevant concepts, (3) linkage of the concepts to the ICF, and (4) frequency analysis. The selected search strategy and methods of analysis of this review were registered in the PROSPERO database (ref: CRD42020156209). This report was written following the guidelines of the Preferred Reporting Items for Systematic reviews and Meta-Analyses Extension for Scoping Reviews (PRISMA-ScR) checklist [14].

### 2.2. Literature Search

An extensive literature search was conducted using the following electronic databases: MEDLINE/PubMed, CINAHL, Web of Science, Scopus, and PEDro. The studies published between January 2012 and June 2022 in English or Spanish were considered for inclusion. Combinations and variations of keywords and medical subject headings were used in each database: musculoskeletal conditions, primary health care, physical therapy, body functions, body structures, activities and participation, environmental factors musculoskeletal disorders, physiotherapy, primary health care, and outcomes measures. The complete search strategy can be found in Appendix A.

### 2.3. Study Selection

Studies were included according to the PICOS framework (population, intervention, comparison, outcomes, study design). To focus on the goal of this review, we did not use “C” as it was not considered relevant.

Population: the participants included in the published study had to be from Western countries (United States of America, Canada, Australia, New Zealand, United Kingdom, European Union, and member countries of the European Free Trade Association, such as Norway or Switzerland), and the sample included people older than 18 years diagnosed with a musculoskeletal condition in a primary care health setting.

Intervention: a physiotherapy intervention in a primary care setting was applied.

Outcomes: the publications had to be related to functioning as defined by the ICF.

Study design: randomized controlled trials, clinical controlled trials, cross-sectional studies, observational studies, and qualitative studies published were included.

Studies were excluded if they were based solely on specific health problems, the sample was not representative of the general population (the study selected participants according to their age, sex, race, nationality, etc.), the study was conducted over hospitalized participants, or the research was a study protocol, a systematic review, a meta-analysis, a case report, a doctoral thesis, a letter, a comment, or an editorial.

Results from the searches were gathered in LibreOffice Calc, and duplicates were removed. In the first round, titles and abstracts were screened for eligibility. Subsequently, full-text articles of the included abstracts were retrieved and screened for eligibility.

Two authors (H.H.L. and S.J.D.B.) screened the titles and abstracts of the identified studies for eligibility. After independently reviewing the selected studies for inclusion, Cohen’s kappa statistic was calculated to measure inter- and intra-rater reliability. If it was not clear whether the study met the inclusion criteria, advice was sought from a third researcher (L.C.L.) and an opinion consensus was formed. Once the agreement was reached, a full-text copy of the selected studies was obtained.

### 2.4. Extraction of Relevant Concepts

Relevant information from the selected studies was gathered using a standardized data collection form designed for this purpose. The items included were (a) the country and region where it was carried out, (b) the research design, (c) the size sample, (d) the participant characteristics (age and condition), and (e) assessment instruments used as outcome measures.

Data were independently extracted by two authors (H.H.L. and S.J.DB.) using the form (above). All discrepancies were reviewed, and an agreement was reached through discussion. In the event of disagreement, a third reviewer (L.C.L.) was consulted.

All assessment instruments used in the included studies were recorded, and the number of studies in which the individual measures were used was documented. Outcome measures were classified following the next criteria: (a) they were single or multi-item (e.g., the visual analogic scale for pain is a single-item measure and the neck disability index is a multi-item measure), (b) they could be patient-oriented measures (e.g., self-report questionnaires), clinical assessment (including those requiring specialized equipment), or non-tool measures (often single-patient-oriented questions).

From the outcome measures, individual items were extracted to be linked to the ICF.

### 2.5. Linkage of the Concepts to the ICF

The linking process consists of translating relevant concepts found in measurement instruments into ICF second-level categories. To achieve this, Cieza’s work was taken as a reference [15], and the WHO eLearning tool (www.icf-elearning.com (accessed on 7 December 2022)) about ICF was also used.

Meaningful concepts were identified from each item extracted from the outcome measures. A concept was defined as one separate meaningful entity; one or more concepts could be identified from a single item. The meaningful concepts were then linked to the most precise ICF category in the components of “body functions”, “body structures”, “activities and participation”, and “environmental factors” (e). Concepts were also linked to “personal factors” (pf), although these are not yet classified in the ICF. In case a concept was too general or vague, the code “nd” was assigned (not definable). Similarly, if the information was beyond the scope of the ICF, code “nc” (not covered) was used.

The linking process was performed independently by the same two reviewers (H.H.L. and S.J.D.B.). Results were compared, and disagreements were resolved by discussion. Discrepancies were discussed with a third reviewer (L.C.L.) until a final agreement was reached. Inter-rater agreement of the independent linking conducted for second-level categories was calculated with Cohen’s kappa.

### 2.6. Frequency Analysis

Frequency analysis was carried out to examine the total number of outcome measures and identified ICF categories. If an ICF category was repeatedly assigned within one multiple-item measure, it was counted only once.

### 2.7. Comparison with the ICF Core Set for Post-Acute Musculoskeletal Conditions

A comparison was made between the ICF categories identified and the comprehensive ICF core set for post-acute musculoskeletal conditions [10]. This ICF core set is composed of 70 ICF categories (7 categories belonging to the component “body structures”, 23 from the ICF component “body functions”, 22 from “activities and participation”, and finally, 18 from “environmental factors”). This ICF core set was used as a reference standard to assess whether the identified outcome measures are adequate to cover the essential aspects of functioning in our target population. The decision to select this ICF core set was made based on their similarity to the target population.

Additional ICF categories were also recorded and were considered relevant if they were identified in 5% or more of the selected studies [13]. Additional ICF categories were defined as those identified in the outcome measures but not included in the ICF core set taken as a reference.

## 3. Results

### 3.1. Study Selection

**Figure 1:**
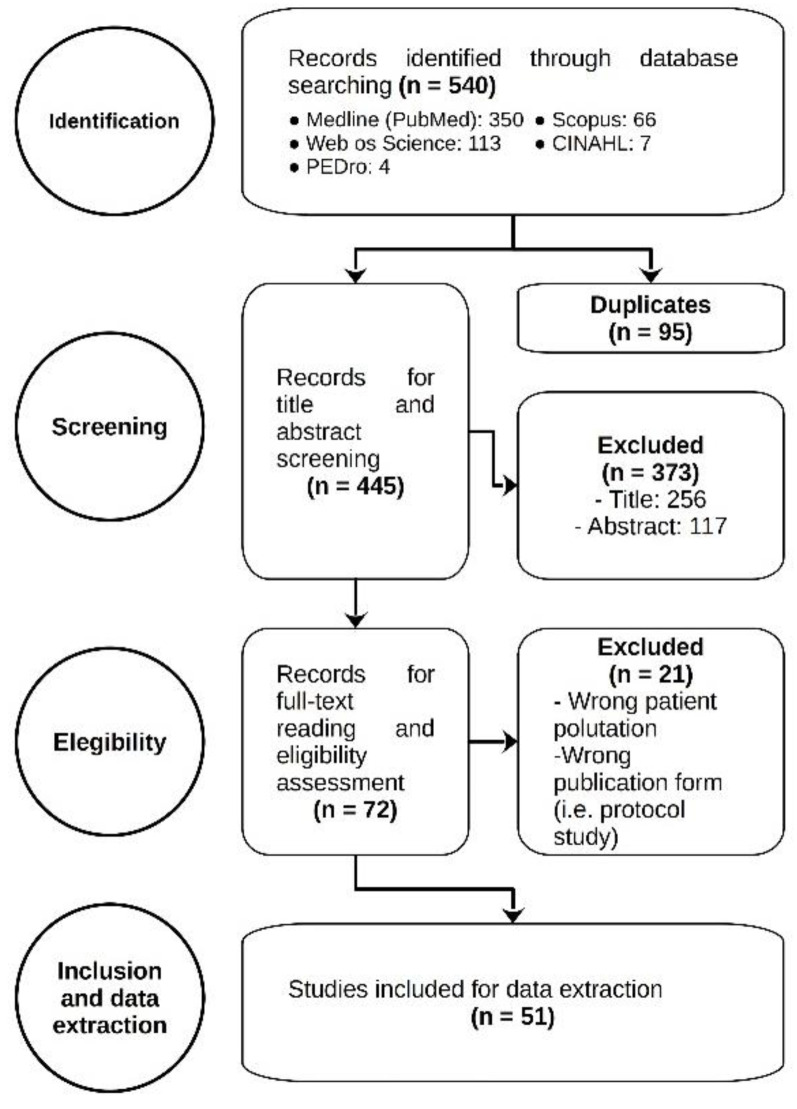
Flowchart diagram.

The search of the scientific literature yielded a total of 540 potentially relevant publications. Ninety-five publications were eliminated because they were duplicates. In the screening process, 256 articles were discarded by title and 117 after reading the abstract. The remaining 72 articles were screened by a full-text reading and 51 were included in the analysis [16,17,18,19,20,21,22,23,24,25,26,27,28,29,30,31,32,33,34,35,36,37,38,39,40,41,42,43,44,45,46,47,48,49,50,51,52,53,54,55,56,57,58,59,60,61,62,63,64,65,66] (Figure 1 shows the flowchart of this process). The Cohen’s kappa coefficient for this process was 0.76 [95% CI: 0.67–0.85].

### 3.2. Study Characteristics

The included studies were conducted in 14 countries. European countries were the most frequent location, accounting for 66.7% of the total (34 studies distributed in the United Kingdom [10]; Norway, Spain, and Sweden [5 each]; Denmark [3]; the Netherlands [2] and Belgium, Germany, Ireland, and Italy [1 each]). Oceania accounted for 17.6% (9 in total, distributed in Australia [6] and New Zealand [3]), and the remaining 15.7% (8) were performed in North America (United States of America [7] and Canada [1]).

The pooled sample size of these studies included a total of 14,702 patients with a musculoskeletal condition. The most studied disorder corresponded to non-specific musculoskeletal pain (such as low back pain, neck pain, or shoulder pain), corresponding to 74.5% of the studies. The next most relevant health problem, accounting for 19.6% of the studies, was degenerative musculoskeletal disorders, such as osteoarthritis of the hip, knee, hand, etc. Finally, 5.9% of the studies focused on specific pain syndromes, such as subacromial syndrome, tennis elbow, and greater trochanteric pain syndrome.

Regarding study design, 38 (74.5%) corresponded to experimental studies, with the randomized controlled trial being the main type (94.7% of all experimental studies). Observational studies accounted for 25.5% of the total (13 studies), and cohort studies were the most frequent design (see Appendix B).

### 3.3. Outcome Measures

A total of 108 assessment instruments were identified from the 51 studies selected. Seventy-four of the outcome measures identified were multi-item (e.g., Oswestry Disability Index), whereas the remaining 34 were single-item (e.g., Visual analog scale) in nature (see Table 1 and Appendix C).

These instruments were classified according to the main aspect of functioning they were intended to assess, the most relevant being the following: (a) disability (28 outcome measures), (b) presence of psychosocial factors (17), (c) pain description (13), (d) physical measures (9), (e) physical performance (9), (f) quality of life (8), (g) global perception of change (2), and (h) others (22). Regarding the outcome measures, the most frequently used in relation to the areas of assessment described above were, respectively: (a) Roland Morris questionnaire (11 studies), (b) fear-avoidance beliefs questionnaire (8), (c) numeric pain rating scale (35), (d) range of motion measure (9), (e) physical activity level measure (3), (f) EuroQoL-5D (12), (g) global rating of change score (9) and (h) indirect measure of recovery (10).

### 3.4. Linking Results

A total of 1129 concepts were extracted from the selected assessment tools. Out of these, 1110 concepts were linked to second-level ICF categories. Nineteen concepts could not be assigned to a specific ICF category due to the concepts being ambiguously defined or beyond the scope of the classification. Linkable concepts were related to 147 ICF categories. The Kappa coefficient for this process was 0.72 [95% CI: 0.65–0.79]. Sixty-two (42.2%) of these categories belonged to the “activities and participation” component, 55 (37.4%) to the “body functions” component, 22 (15.0%) to the “environmental factors” component, and finally, 8 (5.4%) categories from the “body structures” component. The most frequently mentioned category for each ICF component were, respectively, d450 Walking (counted 90 times), b280 Sensation of pain (207), e355 Health professionals and e580 Health services, systems and policies (73 both), and s760 Structure of trunk (33).

Regarding not linkable concepts, 11 of them could not be linked because they corresponded to personal factors (pf) (e.g., age, gender, body mass index, etc.). Four concepts were classified as “nd” due to their ambiguity (e.g., the item “would you accept a handshake without reluctance?” from the functional index for hand arthropathies may lead to multiple interpretations and was not linked to a specific ICF category). Finally, 4 other concepts were related to ICF but did not fit into any category (e.g., adverse events or the number of general practitioner visits).

### 3.5. Comparison with Comprehensive ICF Core Set for Post-Acute Musculoskeletal Conditions

The ICF categories obtained from the concepts of functioning identified in the outcome measures coincide 84.2% with those present in the ICF core set taken as a reference standard. The outcome measures identified in our study were not able to cover eleven categories present in the ICF core set. These categories belonged to the components “environmental factors” (e125 Products and technology for communication, e225 Climate, e410 Individual attitudes of immediate family members, e420 Individual attitudes of friends, e440 Individual attitudes of personal care providers and personal assistants, e555 Associations and organizational services, systems and policies, e575 General social support services, systems and policies), “activities and participation” (d155 Acquiring skills, d310 Communicating with and receiving spoken messages), “body functions” (b435 Immunological system functions) and “body structures” (s810 Structure of areas of skin). Table 2 shows a relation between outcome measures and ICF categories in the brief ICF core set for post-acute musculoskeletal conditions (frequencies for the ICF categories in the comprehensive ICF core set can be found in Appendix D).

A total of 87 additional ICF categories were extracted from the outcome measures analyzed. Sixty-eight of these categories exceeded the 5% threshold and were considered relevant. Forty-one of these categories belonged to the component “activities and participation”, 19 to “body functions”, 7 to “environmental factors”, and 1 to “body structures”. The most relevant additional ICF category for each ICF component were, respectively, d859 Work and employment, other specified and unspecified (identified in 86.3% of the studies), b720 Mobility of bone functions (82.4%), e399 Support and relationships, unspecified (25.5%) and s770 Additional musculoskeletal structures related to movement (5.9%). A full list of additional ICF categories can be found in Appendix E.

## 4. Discussion

This scoping review has identified the most relevant functioning features for the management of musculoskeletal conditions in primary care physiotherapy services from a researcher’s perspective. The aim was to obtain an ICF profile that best fits this specific clinical setting. According to our results, ICF categories belonging to the component “activities and participation” were the most numerous (62 out of 147, 42.2%). However, the most frequent ICF categories belonged to the component “body functions” (e.g., b280 Sensation of pain or b710 Mobility of joint functions were counted 207 and 104 times, respectively).

Pain assessment was considered the most important functional aspect, with up to 13 outcome measures identified for this purpose. Moreover, the outcome measures were not only addressed to the assessment of pain but also to identify features related to its chronification, such as tests to discriminate nociplastic pain (e.g., detection of pain thresholds, temporal summation, or conditional pain modulation) [67]. This finding is in accordance with the multidimensional definition of pain formulated by the International Association for the Study of Pain (IASP) [68] and the recommendations of the Initiative on Methods, Measurement, and Pain Assessment in Clinical Trials (IMMPACT) [69]. It also responds to the significant impact in terms of disability that chronic pain as a clinical entity is having on the world’s population in recent decades [70,71].

The assessment of movement was the second most relevant aspect considered in the outcome measures analyzed. In terms of ICF, movement can be described by means of a broad set of categories. Van Dijk et al. [72] have contributed to clarifying this issue through a study on the quality of movement in patients with low back pain. As these authors have observed, movement is a complex entity that not only includes structural (e.g., joints, muscles, etc.) and functional aspects (e.g., motor control, proprioception, etc.), but it also involves significant mental functions (e.g., insight, motivation, emotions, etc.). The same conclusion can be drawn from the findings of this review since all the second-level categories belonging to the ICF chapter b7 Neuromusculoskeletal and movement-related functions were identified in the outcome measures analyzed. This is particularly relevant because movement is the core expertise of physiotherapy as a profession and it can be concluded that it has a central role in the management of musculoskeletal disorders [73,74]. Moreover, this is consistent with the contribution of Finger et al. in describing within the ICF framework the profile of patients receiving healthcare by physiotherapists [75].

Psychosocial aspects also play an important role in the assessment of musculoskeletal disorders. ICF categories such as b130 energy and drive functions, b152 emotional functions, and b160 thought functions (which includes b1602 content of thought) are among the most frequently identified in the outcome measures used in musculoskeletal research. In the context of this review, these categories can be considered cross-cutting to the concepts of pain and movement described above. Catastrophism, kinesiophobia, and fear-avoidance beliefs are aspects that have been described in the context of chronic pain and can lead to behavioral changes that produce movement disorders. The relationship between pain, function, and psychosocial factors has already been established by some authors [76,77,78], and they are predictors of disability and work absence [79].

Regarding the “activities and participation” component, the categories belonging to the ICF chapters d4 Mobility, d5 Self-care, and d6 Domestic life are widely considered in the assessment instruments. These tools are typically patient-reported outcome measures (PROM), generally oriented to specific pathologies (e.g., neck disability index) or body regions (e.g., DASH). There is controversy in the scientific literature about the validity of such measures [80]. In terms of individual categories, d450 Walking was the most frequently identified. Gait speed has been proposed by some authors as a predictor of disability and quality of life [81,82].

In relation to the “environmental factors” component, a total of 22 ICF categories were identified, but with a substantially lower frequency than the above-mentioned components. Only 6 outcome measures were intended to assess an environmental factor, so the linking process to the ICF was made based on the outcome measures that address these factors indirectly. The most frequently identified aspect was the quality of health care (e.g., through an instrument such as the osteoarthritis quality indicator questionnaire), which was conceptualized as a combination of the following ICF categories: e355 Health professionals, e450 Individual attitudes of health professionals, and e580 Health services, systems and policies. The lack of specific outcome measures to assess environmental factors may be related to the difficulty in conceptualizing this component of the ICF. As Day et al. [83] stated, although the ICF is an advanced framework for describing functional status in relation to health, the current coding system may not be adequate to describe the facilitator–barrier continuum.

Additionally, the information related to the component “body structures” allowed linking all the categories of the ICF chapter s7 Structures related to movement and the ICF category s120 Spinal cord and related structures. However, we cannot consider this finding sufficiently relevant because the frequency for these categories was low. Furthermore, the identification of body regions is based on the target population of the selected studies. For example, the most frequent category was s760 Trunk structure, but this could be due to the fact that 18 studies (35.3%) included patients with low back pain. In our opinion, the ICF category s770 Additional musculoskeletal structures related to movement is more versatile and inclusive for the review purpose, because it considers body structures in a non-specifically manner rather than the other categories in this chapter.

Finally, personal factors were not analyzed in this review because this component has not yet been developed in the ICF. Authors such as Geyh et al. [84] have proposed the opening of a scientific discussion to develop this area and increase the potential of the ICF.

In the comparison with the ICF core set for post-acute musculoskeletal conditions, there was a high percentage of agreement (84.2%) with the ICF categories obtained from the outcome measures. However, assuming without further consideration that there is good coverage of the relevant aspects of functioning can be misleading. The assessment tools that account for the majority of ICF categories are PROMs, and some authors have questioned the content validity of these instruments [85]. In recent years, efforts have been made to improve the properties of these outcome measures [86], but as some authors recommend, caution must be taken in the selection of such tools [87].

Moreover, a large number of additional ICF categories have been identified, so there are several areas of functioning that are considered important from the researcher’s point of view but are not represented in the ICF core set taken as a reference standard. This could be due to the nature of this ICF core set, since it is intended to be used by multidisciplinary teams in rehabilitation facilities [11]. However, primary care teams are not only focused on rehabilitation and they could have specific needs in terms of functioning description. According to the results of our study, there are some poorly covered areas of functioning when the ICF core set for post-acute musculoskeletal conditions is oriented to a primary care context.

Additional ICF categories belonging to the component “activities and participation” were mainly related to chapters d4 Mobility, d6 Domestic life, and d8 Major life areas, including education, employment, and economic life. The most frequent categories were consistent with this finding and d859 Work and employment, other specified and unspecified (included in 86.3% of the studies), d640 Doing housework (72.5%), d920 Recreation and leisure (68.6%), and d455 Moving around (52.9%) were identified. Regarding “body functions”, ICF category b720 Mobility of bone functions was the most frequent (82.4%). The most relevant ICF chapter was d1 Mental functions, including categories such as b180 Experience of self and time functions (74.5%), b160 Thought functions (68.6%), and b126 Temperament and personality functions (49.0%). A broader description of pain seems necessary, taking into account the identification of b289 Sensation of pain, other specified and unspecified (56.9%). Finally, a myriad of “environmental factors” was also identified, but apparently with less relevance and more difficulty in reaching a clear consensus. This is the case for ICF categories e399 Support and relationships, unspecified (25.5%), e570 Social security services, systems and policies (23.5%), or e325 Acquaintances, peers, colleagues, neighbors and community members (17.6%).

In view of the above, the need to develop a tailored core set for primary care should be considered. The existing ICF core sets are adequate to describe the early stages of the rehabilitation process, starting in the acute hospital and continuing in rehabilitation centers [11]. However, there is a lack of a comparable ICF-based tool that can be used in the later stage of the continuum of care, where patients are reintegrated into the community. To some extent, some authors have already pointed to this need by calling for an ICF core set for chronic musculoskeletal conditions [88], which could also be applied in a primary care setting. The availability of a tailored ICF core set has deep implications, as it is the framework that allows the selection of the most appropriate assessment tools for a given clinical context.

Limitations of this study include potential biases arising from study selection, extraction of outcome measures, and those related to the ICF linking process. Regarding the selection of studies, only publications in English and Spanish were selected, so relevant information from studies published in another language may have been missed. The authors decided not to set a threshold for the selection of outcome measures in order to make the analysis as exhaustive as possible. However, this implied analyzing a high number of assessment instruments and resulted in linking ICF categories with very low frequency (e.g., there were 97 categories with a frequency of less than 20). This should be taken into account when interpreting the data. Finally, although there are established rules for the linking process [15], a certain degree of subjectivity on the part of researchers is inevitable. Therefore, the categories linked could be biased in some way.

In summary, the findings of this review provide relevant information about the researcher’s perspective on the most frequent tools used in the assessment of musculoskeletal conditions in a primary care physiotherapy setting. To our knowledge, this is the first study to address this issue in a comprehensive manner. This type of review is usually conducted as part of the preparatory studies carried out during the development of ICF core sets [89,90]. The aim of this exploratory phase is to capture the perspective of researchers, practitioners, patients, and the healthcare context [13]. Therefore, the results of this study not only allow for a better selection of outcome measures in clinical practice but also contribute to laying the foundations for the development of a tailored core set for physiotherapy units in primary care.

## 5. Conclusions

The findings of this study contribute to a better understanding of the most relevant aspects of functioning in the management of patients with musculoskeletal conditions from the researcher’s perspective. This knowledge is potentially useful for the development of ICF-based assessment tools.

## Figures and Tables

**Table 1:** Frequency and thematic focus of assessment instruments included (identified in 4 or more studies).

Assessment Instrument	Main Theme	Type	No of Studies
11-points numeric pain rating scale (NPRS)	Pain description	Single-item	27
0–100 mm. Visual analog scale (VAS)	Pain description	Single-item	11
Question about pain	Pain description	Single-item	7
Örebro Musculoskeletal Pain Screening questionnaire (OMPQ)	Pain description	Multi-item	4
Pressure pain detection threshold (PPT)	Pain description	Single-item	4
Roland Morris Disability questionnaire (RMDQ)	Disability	Multi-item	11
Question about disability	Disability	Single-item	10
Oswestry Disability Index (ODI)	Disability	Multi-item	8
Western Ontario and McMaster Universities Arthritis Index (WOMAC)	Disability	Multi-item	8
Neck disability index (NDI)	Disability	Multi-item	5
Disabilities of the arm, shoulder, and hand (DASH)	Disability	Multi-item	4
Shoulder pain and disability index (SPADI)	Disability	Multi-item	4
Work absence reported	Disability	Single-item	4
Fear avoidance beliefs questionnaire (FABQ).	Psychosocial factors	Multi-item	8
Tampa Scale for Kinesiophobia (TSK)	Psychosocial factors	Multi-item	7
Pain catastrophizing scale (PCS)	Psychosocial factors	Multi-item	4
Question about psychosocial factors	Psychosocial factors	Single-item	4
EuroQoL 5 dimensions (EQ5D)	Quality of life related to health	Multi-item	11
Short form health survey 36 questionnaire (SF-36)	Quality of life related to health	Multi-item	5
Short form health survey 12 questionnaire (SF-12)	Quality of life related to health	Multi-item	4
Global rating of change (GROC)	Global perception of change	Single-item	9
Perceived recovery	Global perception of change	Single-item	7
Range of movement measure (ROM)	Physical measure	Single-item	7
Physical activity level measure	Physical performance	Single-item	7
Variation in the use of analgesics or other therapies	Other (indirect measure of recovery)	Single-item	10
Patient Satisfaction questionnaire	Other (patient satisfaction)	Multi-item	6
Adherence to treatment	Other (personal factor)	Single-item	5
Adverse events reported	Other (adverse events)	Single-item	4

This list continues in Appendix C.

**Table 2:** Comparison with the Brief ICF Core Set for Post-Acute Musculoskeletal Conditions.

ICF Category *	Count	Outcome Measures
b134 Sleep functions	58	PSQI, SQM (actigraphy)
b260 Proprioceptive function	4	Physical performance measures (e.g., SPPB)
b280 Sensation of pain	207	NPRS, VAS, OMPQ, PPDT, TS, CPM, BC, CSI, CPAQ, FABQ, GCPS, PCS, TSK
b435 Immunological system functions	0	-
b530 Weight maintenance functions	11	IPQ, DRAM, PHQ
b620 Urination functions	4	DRAM, CSI
b730 Muscle power functions	104	Physical measure (e.g., dynamometer)
b740 Muscle endurance functions	99	Physical measure (e.g., McQuade test)
b755 Involuntary movement reaction functions	93	Region-specific functional scales (e.g., BPS) or physical performance measures (e.g., TUG)
b780 Sensations related to muscles and movement functions	29	HSC, MHQ, OSPRO-YF, IPQ, CSI, region-specific functional scales (e.g., DASH)
d155 Acquiring skills	0	-
d177 Making decisions	5	DRAM, BDI
d230 Carrying out daily routine	23	Quality of life scale (e.g., SF-36), pain-related questionnaires (e.g., CPAQ or PEI), CSQ, DRAM, MHQ
d240 Handling stress and other psychological demands	41	AQLI, BDI, CSQ, DASS, GAD, OSPRO-YF, PCS, PAM, PHQ, SF-36, STAI
d410 Changing basic body position	46	LEFS, region-specific functional scale (e.g., HOOS)
d415 Maintaining a body position	64	DRI, region-specific functional scales
d430 Lifting and carrying objects	43	DAQ, DRI, PAT5, region-specific functional scales (e.g., ODI or SPADI),
d445 Hand and arm use	16	DASH, FIHOA, OSS, PRTEE, SPADI
d450 Walking	90	TUG, LEFS, ASES, DRI, DAQ, quality of life scales (e.g., EQL5), region-specific functional scale (e.g., KOOS)
d465 Moving around using equipment	29	DAQ, IPAQ, SGPAL
d510 Washing oneself	70	SF-36, AQLI, DRI, EQL5, MHQ, PDI, region-specific functional scale (e.g., DASH)
d520 Caring for body parts	12	AQLI, region-specific functional scale (e.g., DASH)
d530 Toileting	19	AQLI, PAT5, region-specific functional scale (e.g., WOMAC)
d540 Dressing	83	ASES, AQLI, DRI, EQL5, MHQ, PDI, PAT5, region-specific functional scale (e.g., PRTEE)
d550 Eating	18	AQLI, DRAM, PDI, PAT5, region-specific functional scale (e.g., DASH)
e110 Products or substances for personal consumption	36	BBQ, OSPRO-ROS, OCCQ, PAM
e115 Products and technology for personal use in daily living	24	ASES, HUI3
e120 Products and technology for personal indoor and outdoor mobility and transportation	37	BBQ, HUI3, OA-QI
e225 Climate	0	-
e355 Health professionals	73	AdEv, ARM, BBQ, CSQ, ECRQ, MRI, OA-QI
e450 Individual attitudes of health professionals	63	CSQ, ECRQ, MRI, OA-QI, PSEQ

* Initial letters show ICF component (“b” for “body functions”; “d” for “activities and participation”, “e” for “environmental factors” and “s” for “body structures”). AdEv: adverse events reported; AQLI: assessment quality of life instrument; ARM: attitudes regarding responsibility for musculoskeletal disorders scale; ASES: arthritis self-efficacy scale; BBQ: Back Beliefs questionnaire; BDI: Beck Depression Inventory; BPS: back performance scale; BC: Bournemouth Questionnaire; CPAQ: chronic pain acceptance questionnaire; CPM: conditional pain modulation; CSI: central sensitization inventory; CSQ: coping strategies questionnaire; DAQ: daily activities questionnaire; DASH; disability of arm shoulder and hand; DASS: depression anxiety stress scale; DRAM: distress and risk assessment method; DRI: disability rating index; ECRQ: effective consultation and reassurance questionnaire; EQL5: Euro quality of life-5D; FABQ: fear avoidance beliefs questionnaire; FIHOA: functional index for hand ostheoarthritis; GAD: generalized anxiety disorder questionnaire; GCPS: graded chronic pain scale; HOOS: hip disability and osteoarthritis outcome score; HSC: Hopkins symptoms checklist; HUI3: health utilities index-3; IPAQ: international physical activity questionnaire; IPQ: illness perception questionnaire; KOOS: knee injury and osteoarthritis outcome score; LEFS: lower extremity functional scale; MHQ: musculoskeletal health questionnaire; MRI: MedRisk instrument; NPRS: numeric pain rating scale; OA-QI: quality indicators for the management of ostheoarthritis; OCCQ: Otago Costs and Consequences questionnaire for low back pain; ODI: Oswestry Disability Index; OMPQ: Örebro Musculoskeletal Pain Screening questionnaire; OSPRO-ROS: OSPRO Review of Systems tool; OSPRO-YF: OSPRO Yellow Flag tool; OSS: Oxford shoulder scale; PAM: patient activation measure; PAT5: paper adaptative test-5D; PCS: pain catastrophizing scale; PDI: pain disability index; PEI: pain enablement instrument; PHQ: patient health questionnaire; PPT: pressure pain threshold; PQ: pain question; PR: perceived recovery; PRTEE: patient-reported tennis elbow evaluation; PSEQ: pain self-efficacy questionnaire; PSQI: Pittsburgh Sleep Quality Index; SF-36: 36-item short form survey; SGPAL: Saltin–Grimby physical activity level scale; SPADI: shoulder pain and disability index; SPPB: short physical performance battery; SQM: sleep quality measure; STAI: state trait anxiety inventory; TS: temporal summation; TSK: Tampa Scale for Kinesiophobia; TUG: timed-up and go; VAS: visual analog scale; WOMAC: Western Ontario McMaster Universities Osteoarthritis Index.

## Appendix A. Search Strategy

**Database:** Medline/Pubmed

**Search strategies:**

1. ((("Musculoskeletal Diseases"[Mesh] AND "Primary Health Care"[Mesh])) AND "Physical Therapy Modalities"[Mesh]) AND ("Outcome Assessment, Health Care"[Mesh] OR "Patient Reported Outcome Measures"[Mesh] OR "International Classification of Functioning, Disability and Health"[Mesh])
2. (((musculoskeletal AND (disease* OR condition* OR disorder*)) AND (primary health care OR Community-Based Primary Care)) AND (physical therapy modalities OR physical therapy OR physiotherapy)) AND (body function* OR body structure* OR activit* OR participation* OR ICF OR international classification of functioning disability and health OR outcomes measures)

**Filters applied:** article type (Clinical Study, Clinical Trial, Comparative study, Controlled Clinical Trial, Multicenter Study, Observational Study, Pragmatic Clinical Trial, Randomized Controlled Trial), publication date (last 10 years), language (english, spanish)

**Database:** Scopus

**Search strategies:**

- musculoskeletal condition AND physiotherapy AND primary health care AND outcomes measures (title-abs-key)
- musculoskeletal disorder AND physiotherapy AND primary health care AND outcomes measures (title-abs-key)
- musculoskeletal condition AND physical therapy AND primary health care AND outcomes measures (title-abs-key)
- musculoskeletal disorder AND physical therapy AND primary health care AND outcomes measures (title-abs-key)

**Filters applied:** document type (article), language (english), year (from 2012).

**Database:** CINAHL

**Search strategies:**

1. musculoskeletal condition AND physiotherapy AND primary health care AND outcomes measures
2. musculoskeletal disorder AND physiotherapy AND primary health care AND outcomes measures
3. musculoskeletal condition AND physical therapy AND primary health care AND outcomes measures
4. musculoskeletal disorder AND physical therapy AND primary health care AND outcomes measures

**Filters applied:** publication date (from 2012)

**Database:** Web of Science

**Search strategies:**

- musculoskeletal condition AND physiotherapy AND primary health care AND outcomes measures
- musculoskeletal disorder AND physiotherapy AND primary health care AND outcomes measures
- musculoskeletal condition AND physical therapy AND primary health care AND outcomes measures
- musculoskeletal disorder AND physical therapy AND primary health care AND outcomes measures

**Filters applied:** publication date (from 2012)

**Database:** PEDro

**Search strategies:**

1. musculoskeletal disorder physical therapy primary health care outcomes measures
2. musculoskeletal disorder physiotherapy primary health care outcomes measures
3. musculoskeletal condition physical therapy primary health care outcomes measures
4. musculoskeletal condition physiotherapy primary health care outcomes measures

## Appendix B

**Table A1:** Characteristics of Included Studies.

Study	Country	Design	Sample Size	Participants			Outcome Measures *
				Age (Years)	Female/Male	Pathology	
Abbot et al. (2019)	New Zealand	Experimental (RCT)	206	37-92	114/92	Hip or knee osteoarthritis	Primary: WOMAC Secondary: NPRS, WT, STS, TUG, AdEv
Allen et al. (2017)	United States of America	Experimental (RCT)	537	NR	397/140	Hip or knee osteoarthritis	Primary: WOMAC Secondary: PHQ, SPPB, ATU, PAL
Amorim et al. (2019)	Australia	Experimental (RCT)	68	>18	34/34	Chronic low back pain	Primary: CS, NPRS, RMDQ Secondary: PAL, DASS, FABQ, IPAQ, PSQI
Arden et al. (2017)	United Kingdom	Observational (RCS)	62	>18	39/23	Low back pain	Primary: BQ, WT, ST, STS
Battista et al. (2021)	Italy	Observational (DQS)	11	NR	6/5	Hip and knee osteoarthritis	Primary: DQ (3)
Benell et al. (2017)	Australia	Experimental (RCT)	148	>50	83/65	Chronic Knee Pain	Primary: NPRS, WOMAC Secondary: GROC, PCS, AQLI, ASES, CSQ, AdEv
Benell et al. (2014)	Australia	Experimental (RCT)	78	NR	42/36	Knee osteoarthritis	Primary: VAS, WOMAC Secondary: Adh
Bornhöft et al. (2019)	Sweden	Experimental (RCT)	55	16-67	34/21	Musculoskeletal disorders	Primary: NPRS, DRI, EQL5, OMPQ Secondary: ARM
Burns et al. (2018)	United States of America	Experimental (RCT)	90	≥18	37/53	Low back pain	Primary: NPRS, ODI, GROC
Chesterton et al. (2013)	United Kingdom	Experimental (RCT)	241	NR	109/132	Tennis elbow	Primary: NPRS Secondary: GROC, PRTEE, EQL5, IPQ, SF-12
Christiansen et al. (2018)	Denmark	Observational (PCS)	160	>18	90/70	Neck, shoulder, and low-back pain	Primary: DASH, NPRS, NDI, OMPQ, RMDQ, WHO5
Costa et al. (2022)	Portugal	Experimental (NCIS)	343	>18	205/138	Musculoskeletal pain	Primary: NPRS Secondary: ATU (2), GAD, PHQ, FABQ, WPAI, Adh
Crossley et al. (2015)	Australia	Experimental (RCT)	92	>40	53/39	Patelofemoral osteoarthritis	Primary: GROC, KOOS, VAS Secondary: Adh, AdEv
Cuesta-Vargas et al. (2015)	Spain	Experimental (RCT)	114	NR	NR	Chronic musculoskeletal disorders	Primary: SF-12, EQL5, VAS, RMDQ, NDI, WOMAC
Darlow et al. (2019)	New Zealand	Experimental (RCT)	221	NR	105/116	Low back pain	Primary: RMDQ Secondary: NPRS, DRS, PS, EQL5, OCCQ, PSEQ, PyScFQ (4)
Emilson et al. (2017)	Sweden	Experimental (RCT)	43	18-65	30/10/22	Musculoskeletal pain	Primary: NPRS, PDI, TSK, PR
Ferrer-Peña et al. (2019)	Spain	Observational (CSS)	49	NR	41/8	Greater trochanteric pain syndrome	Primary: PPSA, GCPS, PPT, TS, CPMI, VAS
Gohir et al (2021)	United Kingdom	Experimental (RCT)	105	>45	71/34	Knee osteoarthritis	Primary: NPRS Secondary: WOMAC, STS, TUG, MHQ, MVC, PPT, TS, CPM, SQM, PSQI, MUA
Goldberg et al. (2018)	United States of America	Observational (CSS)	853	>18	458/395	Musculoskeletal pain	Primary: TSK, SF-8
Hill et al. (2020)	United Kingdom	Experimental (RCT)	524	NR	318/206	Musculoskeletal pain (back, neck, knee or multi-site pain)	Primary: RMDQ, NDI, SPADI, KOOS, SF-12 Secondary: STMT, MHQ, TSK, ECRQ, EQL5, PS, GROC, WA, WP, PQ
Hopewell et al. (2021)	United Kingdom	Experimental (RCT)	708	>18	349/359	A rotator cuff disorder	Primary: SPADI Secondary: EQL5
Laslett et al. (2014)	New Zealand	Observational (PCS)	161	>18–81	82/79	Shoulder pain	Primary: SPADI, VAS, FABQ, SF-8, DRS
Leaver et al. (2013)	Australia	Observational (PCS)	181	18-70	117/64	Cervical pain	Primary: PR Secondary: NPRS, NDI
Leemans et al. (2021)	Belgium	Experimental (RCT)	50	25-80	27/23	Low back pain	Primary: NPRS, BPS Secondary: PPT, TS, CPM, FABQ, SF-36, CSI, ATU
Legha et al. (2020)	United Kingdom	Experimental (RCT)	1083	NR	619/464	Knee osteoarthritis	Primary: WOMAC
Lentz et al. (2018)	United States of America	Observational (PCS)	440	NR	275/164	Neck, low back, knee or shoulder	Primary: PQ (2), NPRS, NDI, ODI, DASH, IKDF, OSPRO-ROS, OSPRO-YF
Lewis et al. (2017)	United Kingdom	Experimental (RCT)	227	>18	109/118	Subacromial pain syndrome	Primary: OSS Secondary: SPADI, VAS, DVAS, PQ, ROM, OT-NS, OT-HT
Lingner et al. (2018)	Germany	Experimental (RCT)	87	18-50	44/43	Low back pain	Primary: NPRS, VAS Secondary: ATU (3), HFAQ, GROC, WA, PS
López-López et al. (2015)	Spain	Experimental (RCT)	48	18-65	42/6	Chronic neck pain	Primary: VAS Secondary: ROM, PPT, STAI, BDI, TSK, PCS
Marra et al. (2012)	Canada	Experimental (RCT)	139	≥ 50	79/60	Knee osteoarthritis	Primary: OA-QI Secondary: HUI3, LEFS, PAT5, WOMAC
Matarán-Peñarrocha et al. (2020)	Spain	Experimental (RCT)	64	18-65	32/32	Chronic non specific low back pain	Primary: MQ-OT, FTFd, ODI, RMDQ, TSK, VAS
Miedema et al. (2016)	Netherlands	Observational (PCS)	682	18-64	286/396	Musculoskeletal pain of arm, neck and shoulder	Primary: DASH, PR
Minns Lowe et al. (2020)	United Kingdom	Experimental (RCT)	41	>18	20/21	Musculoskeletal disorders	Primary: WT Secondary: PAL (2), NPRS, PANAS, GSES, SF-36 (1), PR, DAQ
Molgaard Nielsen et al. (2017)	Denmark	Observational (PCS)	928	18-65	418/510	Low back pain	Primary: NPRS, RMDQ Secondary: PQ (3)
Moseng et al. (2020)	Norway	Experimental (RCT)	393	≥45	280/113	Hip and/or knee osteoarthritis	Primary: NPRS, DQ (2), ROM, HOOS, KOOS
Murphy et al. (2013)	Ireland	Observational (PCS)	1532	NR	958/574	Low back pain	Primary: VAS, RMDQ, DRAM, BBQ, ROM, MSPQ, SFAT
Noblet et al. (2020)	England	Experimental (RCT)	29	>18	17/12/22	Low back pain	Primary: NPRS, RMDQ Secondary: EQL5, TSK, PAL, WA, ATU (2)
Østerås et al. (2014)	Norway	Experimental (RCT)	130	40-79	117/13	Hand osteoarthritis	Primary: FIHOA, NPRS, PSFS, DQ Secondary: ROM, GROC, DQ, MVC, MPU-OT, Adh, AdEv
Østerås et al. (2019)	Norway	Experimental (RCT)	393	≥45	279/114	Hip and/or knee osteoarthritis	Primary: OA-QI Secondary: PS, PAL, PR
Paanalahti et al. (2016)	Sweden	Experimental (RCT)	1057	18–65	740/317	Neck pain and/or back pain	Primary: CPQ, NPRS, DQ (3) Secondary: PR, ATU
Palacín-Marín et al. (2013)	Spain	Experimental (RCT)	15	>18	06/09	Lumbar pain	Primary: ROM, ST-OT, SLR-OT, ODI, VAS, SF-12, TSK
Sandal et al. (2021)	Denmark	Experimental (RCT)	461	>18	255/206	Low back pain	Primary: RMDQ Secondary: NPRS, PSEQ, FABQ, IPQ, EQL5, GROC, SGPAL
Schroder et al. (2021)	Sweden	Experimental (RCT)	467	18-65	204/263	Low back pain	Primary: NPRS, ODI Secondary: IPQ, EQL5, PEI, GROC, PS
Schuetze et al. (2014)	Australia	Experimental (NCIS)	16	18-65	12/04/22	Low back pain	Primary: OMPQ, ODI, DASS, MAAS, PCS, CPAQ, SF-36, ClSQ
Trulsson Schouenborg et al. (2021)	Sweden	Observational (PCS)	274	>18	194/80	Chronic musculoskeletal pain	Primary: NPRS Secondary: DRI, EQL5
Uhl et al. (2017)	United States of America	Observational (RCS)	128	NR	74/53	Shoulder pain	Primary: PQ, NPRS, Adh, ATU, DASH
Van der Maas et al. (2015)	Netherlands	Experimental (RCT)	94	NR	77/17	Chronic musculoskeletal pain	Primary: NPRS, BDI, SF-36, PDI, SBC, PSEQ, PCS
Vibe Fersum et al. (2019)	Norway	Experimental (RCT)	121	18-65	33/88	Non-specific low back pain	Primary: OMPQ Secondary: ODI, HSC, FABQ
Vibe Fersum et al. (2013)	Norway	Experimental (RCT)	121	18-65	63/58	Non-specific low back pain	Primary: NPRS, ODI Secondary: HSC, FABQ, ROM, PS, WA, CS
Williams et al (2019)	United Kingdom	Experimental (RCT)	440	>18	288/152	Musculoskeletal disorders	Primary: PSFS Secondary: EQL5, PAM, MRI
Xia et al. (2016)	United States of America	Experimental (RCT)	192	21-54	88/104	Low back pain	Primary: RMDQ Secondary: VAS, FABQ, SF-36

RCT: randomized controlled trial; NCIS: not-controlled interventional study; RCS: retrospective cohort study; PCS: prospective cohort study; DQS: descriptive qualitative study; CSS: cross-sectional study; NR: not reported.* Abbreviations for assessment instruments: AdEv: adverse events reported; Adh: adherence to treatment; AQLI: assessment quality of life instrument; ARM: Attitudes regarding Responsibility for Musculoskeletal disorders scale; ASES: arthritis self-efficacy scale; ATU: analgesic and other therapies usage; BBQ: back beliefs questionnaire; BDI: Beck depression inventory; BPS: back performance scale; BQ: Bournemouth questionnaire; ClSQ: client satisfaction questionnaire; CPAQ: chronic pain acceptance questionnaire; CPM: conditional pain modulation; CPMI: conditioned pain modulation index; CPQ: chronic pain questionnaire; CS: care seeking; CSI: central sensitization inventory; CSQ: coping strategies questionnaire; DAQ: daily activities questionnaire; DASH: Disability of Arm Shoulder and Hand; DASS: depression anxiety stress scale; DQ: disability question; DRAM: distress and risk assessment method; DRI: disability rating index; DRS: disability rating scale; DVAS: visual analog scale for disability; ECRQ: Effective consultation and reassurance questionnaire; EQL5: Euro quality of life-5D; FABQ: fear avoidance beliefs questionnaire; FIHOA: functional index for hand ostheoarthritis; FTFd: finger to floor distance; GAD: generalized anxiety disorder questionnaire; GCPS: graded chronic pain scale; GROC: global rating of change; GSES: general self-efficacy scale; HFAQ: Hannover functional ability questionnaire; HOOS: hip disability and osteoarthritis outcome score; HSC: Hopkins symptoms checklist; HUI3: health utilities index-3; IKDF: International Knee Documentation Committee Subjective Knee Form; IPAQ: International physical activity questionnaire; IPQ: illness perception questionnaire; KOOS: Knee injury and osteoarthritis outcome score; LEFS: lower extremity functional scale; MAAS: mindful attention awareness scale; MHQ: musculoskeletal health questionnaire; MPU-OT: Mobert pick-up test; MQ-OT: McQuade orthopaedic test; MRI: MedRisk instrument; MSPQ: Modified somatic perception questionnaire; MUA: musculoskeletal ultrasonographic assessment; MVC: maximum voluntary contraction; NDI: neck disability index; NPRS: numeric pain rating scale; OA-QI: Quality indicators for the management of ostheoarthritis; OCCQ: Otago costs and consequences questionnaire for low back pain; ODI: Oswestry disability index; OMPQ: Örebro musculoskeletal pain screening questionnaire; OSPRO-ROS: OSPRO Review of Systems tool; OSPRO-YF: OSPRO Yellow Flag tool; OSS: Oxford shoulder scale; OT-NS: Neer sign orthopaedic test; OT-HT: Hawkins’s orthopaedic test; PAL: physical activity level; PAM: patient activation measure; PANAS: positive and negative affect schedule; PAT5: paper adaptative test-5D; PCS: pain catastrophizing scale; PDI: pain disability index; PEI: pain enablement instrument; PHQ: patient health questionnaire; PPSA: percentage pain surface area; PPT: pressure pain threshold; PQ: pain question; PR: perceived recovery; PRTEE: patient-reported tennis elbow evaluation; PS: patient satisfaction; PSEQ: Pain self-efficacy questionnaire; PSFS: patient-specific functional scale; PyScFQ: psychosocial factors question; PSQI: Pittsburgh sleep quality index; RMDQ: Roland Morris disability questionnaire; ROM: range of motion; SBC: scale of bdy connection; SF-8: 8-item short form survey; SF-12: 12-item short form survey; SF-36: 36-item short form survey; SFAT: Simmond’s functional assessment tool; SGPAL: Saltin-Grimby physical activity level scale; SLR-OT: straight leg raise orthopaedic test; SPADI: Shoulder Pain and Disability Index; SPPB: short physical performance battery; SQM: sleep quality measure; ST: step test; ST-OT: Sorensen orthopaedic test; STAI: state trait anxiety inventory; STMT: STarT-MSK tool; STS: sit-to-stand test; TC: treatment change; TS: temporal summation; TSK: Tampa kinesiophobia scale; TUG: timed-up and go; VAS: visual analog scale; WA: work absence; WHO5: WHO-5 well being index; WOMAC: Western Ontario McMaster Universities osteoarthritis index; WP: work productivity; WPAI: work productivity and activity impairment questionnaire; WT: walking test

## Appendix C

**Table A2:** Supplementary List of Assessment Instruments (Identified in 3 or Less Studies).

Assessment Instrument	No of Studies	Type	Main Theme
Illness Perception Questionnaire	3	Multi-item	Other
Knee injury and Osteoarthritis Outcome Score (KOOS)	3	Multi-item	Disability
Pain self-efficacy questionnaire (PSEQ)	3	Multi-item	Disability
Sit-to-stand test	3	Single-item	Physical performance
Temporal summation	3	Single-item	Pain description
Walking test	3	Single-item	Physical performance
Beck Depression Inventory	2	Multi-item	Psychosocial factors
Care seeking	2	Single-item	Other (indirect recovery)
Conditional pain modulation	2	Single-item	Pain description
Depression Anxiety Stress Scales (DASS)	2	Multi-item	Psychosocial factors
Disability Rating Index (DRI)	2	Multi-item	Disability
Disability Rating Scale	2	Single-item	Disability
Hopkins Symptoms Checklist	2	Multi-item	Psychosocial factors
Musculoskeletal Health Questionnaire	2	Multi-item	Disability
OsteoArthritis Quality Indicator questionnaire	2	Multi-item	Other (environmental factor)
Pain Disability Index	2	Multi-item	Disability
Patient Health Questionnaire (PHQ)	2	Multi-item	Psychosocial factors
Patient-Specific Function Scale (PSFS)	2	Multi-item	Disability
Peak muscle strength	2	Single-item	Physical measure
Pittsburgh Sleep Quality Index	2	Multi-item	Other (sleep)
Short Form Health Survey 8 questionnaire (SF-8)	2	Multi-item	Quality of life related to health
Timed up and go (TUG)	2	Single-item	Physical performance
Arthritis Self-Efficacy Scale	1	Multi-item	Disability
Assessment Quality of Life Instrument (AQLI)	1	Multi-item	Quality of life related to health
Attitudes regarding Responsibility for Musculoskeletal disorders scale (ARM)	1	Multi-item	Other (environmental factor)
Back Beliefs Questionnaire (BBQ)	1	Multi-item	Psychosocial factors
Back Performance Scale (BPS)	1	Multi-item	Disability
Bournemouth Questionnaire	1	Multi-item	Pain description
Central Sensitization Inventory (CSI)	1	Multi-item	Pain description
Chronic Pain Acceptance Questionnaire (CPAQ)	1	Multi-item	Psychosocial factors
Chronic Pain Assessment Questionnaire (CPQ)	1	Multi-item	Pain description
Client Satisfaction Questionnaire (CSQ)	1	Multi-item	Other
Conditioned Pain Modulation Index (CPMI)	1	Multi-item	Pain description
Coping Strategies Questionnaire	1	Multi-item	Psychosocial factors
Daily Activities Questionnaire	1	Multi-item	Disability
Disability Visual Analog Scale	1	Single-item	Disability
Distress and Risk Assessment Method (DRAM)	1	Multi-item	Psychosocial factors
Effective Consultation and Reassurance Questionnaire (ECRQ)	1	Multi-item	Other (environmental factor)
Finger-to-floor distance	1	Single-item	Physical measure
Functional Index for Hand OsteoArthritis	1	Multi-item	Disability
General Self-Efficacy Scale	1	Multi-item	Disability
Generalized Anxiety Disorder (GAD)	1	Multi-item	Psychosocial factors
Graded Chronic Pain Scale (GCPS)	1	Multi-item	Pain description
Hannover functional ability questionnaire (FfbH-R)	1	Multi-item	Disability
Hawkin’s test	1	Single-item	Physical measure (orthopaedic)
Health Utilities Index Mark 3 (HUI3)	1	Multi-item	Quality of life related to health
Hip disability and Osteoarthritis Outcome Score (HOOS)	1	Multi-item	Disability
International Knee Documentation Committee Subjective Knee Form (IKDC)	1	Multi-item	Disability
International Physical Activity Questionnaire	1	Multi-item	Physical performance
Lower Extremities Function Scale (LEFS)	1	Multi-item	Disability
McQuade test	1	Single-item	Physical measure (orthopaedic)
MedRisk instrument	1	Multi-item	Other (environmental factor)
Mindful Attention Awareness Scale (MAAS)	1	Multi-item	Other (self-perception)
Moberg Pick-up Test	1	Single-item	Physical measure (orthopaedic)
Modified somatic perception questionnaire	1	Multi-item	Other (self-perception)
Musculoskeletal ultrasonographic assessment	1	Single-item	Other
Neer sign	1	Single-item	Physical measure (orthopaedic)
OSPRO Review of Systems tool (OSPRO-ROS)	1	Multi-item	Psychosocial factors
OSPRO Yellow Flag tool (OSPRO-YF)	1	Multi-item	Psychosocial factors
Otago Costs and Consequences Questionnaire for Low Back Pain	1	Multi-item	Other (environmental factor)
Oxford Shoulder Score	1	Multi-item	Disability
Pain Enablement Instrument	1	Multi-item	Other (self-management)
Paper Adaptive Test-5D (PAT- 5D)	1	Multi-item	Quality of life related to health
Patient Activation Measure	1	Multi-item	Other (self-management )
Patient-rated Tennis Elbow Evaluation (PRTEE)	1	Multi-item	Disability
Percentage Pain Surface Area (PPSA)	1	Single-item	Pain description
Positive and negative affect schedule (PANAS scale)	1	Multi-item	Psychosocial factors
Question about work productivity	1	Single-item	Other (environmental factor)
Saltin-Grimby Physical Activity Level Scale	1	Multi-item	Physical performance
Scale of Body Connection (SBC)	1	Multi-item	Other (self-perception)
Short Physical Performance Battery (SPPB)	1	Multi-item	Physical performance
Simmond’s functional assessment tool	1	Multi-item	Physical performance
Sleep quality measure	1	Single-item	Other (sleep)
Sorensen test	1	Single-item	Physical measure (orthopaedic)
StarT MSK tool	1	Multi-item	Disability
State Trait Anxiety Inventory (STAI)	1	Multi-item	Psychosocial factors
Step test	1	Single-item	Physical performance
Straight leg raise	1	Single-item	Physical measure (orthopaedic)
WHO 5 Well-being Index	1	Multi-item	Quality of life related to health
Work Productivity and Activity Impairment (WPAI)	1	Multi-item	Other (environmental factor)

## Appendix D

**Table A3:** Comparison with the Comprehensive ICF Core Set for Post-Acute Musculoskeletal Conditions.

ICF Category	ICF Chapter (Theme) *	Count
b130 Energy and drive functions	b1 Mental functions (global mental functions)	73
b134 Sleep functions (B)	b1 ^a, b^	58
b152 Emotional functions	b1 Mental functions (specific mental functions)	104
b260 Proprioceptive function (B)	b2 Sensory functions and pain (additional sensory functions)	4
b270 Sensory functions related to temperature and other stimuli	b2 Sensory functions and pain ^b^	3
b280 Sensation of pain (B)	b2 Sensory functions and pain (pain)	207
b415 Blood vessel functions	b4 Functions of the cardiovascular, haematological, immunological and respiratory systems (functions of the cardiovascular system)	1
b435 Immunological system functions (B)	b4^a^ (functions of the haematological and immunological systems)	0
b440 Respiration functions	b4 ^a^ (functions of the respiratory system)	11
b455 Exercise tolerance functions	b4 ^a^ (additional functions and sensations of the cardiovascular and respiratory systems)	45
b525 Defecation functions	b5 Functions of the digestive, metabolic and endocrine systems (functions related to the digestive system)	2
b530 Weight maintenance functions (B)	b5 ^a,b^	11
b620 Urination functions (B)	b6 Genitourinary and reproductive functions (urinary functions)	4
b710 Mobility of joint functions	b7 Neuromusculoskeletal and movement-related functions (functions of the joints and bones)	104
b715 Stability of joint functions	b7 ^a,b^	92
b730 Muscle power functions (B)	b7^a^ (muscle functions)	104
b735 Muscle tone functions	b7 ^a,b^	99
b740 Muscle endurance functions (B)	b7 ^a.b^	99
b755 Involuntary movement reaction functions (B)	b7 ^a^ (movement functions)	93
b760 Control of voluntary movement functions	b7 ^a,b^	94
b770 Gait pattern functions	b7 ^a,b^	66
b780 Sensations related to muscles and movement functions (B)	b7 ^a,b^	29
b810 Protective functions of the skin	b8 Functions of the skin and related structures (functions of the skin)	1
d155 Acquiring skills (B)	d1 ^a^ (basic learning)	0
d177 Making decisions (B)	d1 ^a^ (applying knowledge)	5
d230 Carrying out daily routine (B)	d2 General tasks and demands	23
d240 Handling stress and other psychological demands (B)	d2 ^a^	41
d310 Communicating with - receiving - spoken messages	d3 Communication (communicating with – receiving – spoken messages)	0
d410 Changing basic body position (B)	d4 Mobility (changing and maintaining body position)	46
d415 Maintaining a body position (B)	d4 ^a,b^	64
d420 Transferring oneself	d4 ^a,b^	13
d430 Lifting and carrying objects (B)	d4 Mobility (carrying, moving and handling objects)	43
d440 Fine hand use	d4 ^a,b^	32
d445 Hand and arm use (B)	d4 ^a,b^	16
d450 Walking (B)	d4 Mobility (walking and moving)	90
d460 Moving around in different locations	d4 ^a,b^	44
d465 Moving around using equipment (B)	d4 ^a,b^	29
d510 Washing oneself (B)	d5 Self-care (theme not available)	70
d520 Caring for body parts (B)	d5 ^a,b^	12
d530 Toileting (B)	d5 ^a,b^	19
d540 Dressing (B)	d5 ^a,b^	83
d550 Eating (B)	d5 ^a,b^	18
d560 Drinking	d5 ^a,b^	18
d570 Looking after one’s health	d5 ^a,b^	49
d760 Family relationships	d7 Interpersonal interactions and relationships (particular interpersonal interactions)	48
e110 Products or substances for personal consumption (B)	e1 Products and technology (theme not available)	36
e115 Products and technology for personal use in daily living (B)	e1 ^a,b^	24
e120 Products and technology for personal indoor and outdoor mobility and transportation (B)	e1 ^a,b^	37
e125 Products and technology for communication	e1 ^a,b^	0
e150 Design, construction and building products and technology of buildings for public use	e1 ^a,b^	1
e225 Climate (B)	e2 Natural environment and human-made changes to environment (theme not available)	0
e310 Immediate family	e3 Support and relationships (theme not available)	13
e320 Friends	e3 ^a,b^	14
e340 Personal care providers and personal assistants	e3 ^a,b^	14
e355 Health professionals (B)	e3 ^a,b^	73
e410 Individual attitudes of immediate family members	e4 Attitudes (theme not available)	0
e420 Individual attitudes of friends	e4 ^a,b^	0
e430 Individual attitudes of people in positions of authority	e4 ^a,b^	6
e440 Individual attitudes of personal care providers and personal assistants	e4 ^a,b^	0
e450 Individual attitudes of health professionals (B)	e4 ^a,b^	63
e555 Associations and organizational services, systems and policies	e5 Services, systems and policies (theme not available)	0
e575 General social support services, systems and policies	e5 ^a,b^	0
e580 Health services, systems and policies	e5 ^a,b^	73
s710 Structure of head and neck region	s7 Structures related to movement	8
s720 Structure of shoulder region	s7 ^a^	19
s730 Structure of upper extremity	s7 ^a^	12
s740 Structure of pelvic region	s7 ^a^	1
s750 Structure of lower extremity	s7 ^a^	15
s760 Structure of trunk	s7 ^a^	33
s810 Structure of areas of skin	s8 Skin and related structures	0

* Initial letters show ICF component (“b” for “body functions”; “d” for “activities and participation”, “e” for “environmental factors” and “s” for “body structures”). (B) indicates that the category also belongs to the brief version of the ICF Core Set. ^a^ Same chapter as category above. ^b^ Same theme as category above.

## Appendix E

**Table A4:** Additional ICF Categories (Second-Level) Linked to Concepts Identified in the Assessment Instruments.

ICF code	Description	Count	Included in studies (%)
d859	Work and employment, other specified and unspecified	89	86.3
b720	Mobility of bone functions	93	82.4
b180	Experience of self and time functions	82	74.5
d640	Doing housework	54	72.5
b160	Thought functions	70	68.6
d920	Recreation and leisure	65	68.6
d455	Moving around	44	52.9
d649	Household tasks, other specified and unspecified	40	51.0
b126	Temperament and personality functions	42	49.0
d299	General tasks and demands, unspecified	32	45.1
d910	Community life	37	41.2
d620	Acquisition of goods and services	28	37.3
d999	Community, social and civic life, unspecified	38	35.3
d160	Focusing attention	14	33.3
b289	Sensation of pain, unspecified Sensation of pain, other specified (conditional pain modulation) Sensation of pain, other specified (temporal summation)	23 3 3	29.4 5.9 5.9
d899	Major life areas, unspecified	33	25.5
d770	Intimate relationships	17	25.5
e399	Support and relationships, unspecified	14	25.5
e570	Social security services, systems and policies	23	23.5
d429	Changing and maintaining body position, other specified and unspecified	19	23.5
b140	Attention functions	16	23.5
d159	Basic learning, other specified and unspecified	5	23.5
d699	Domestic life, unspecified	25	21.6
d850	Remunerative employment	18	21.6
d750	Informal social relationships	14	21.6
d650	Caring for household objects	12	21.6
d163	Thinking	18	19.6
d740	Formal relationships	11	19.6
d599	Self-care, unspecified	10	19.6
e325	Acquaintances, peers, colleagues, neighbours and community members	11	17.6
d630	Preparing meals	11	15.7
b122	Global psychosocial functions	10	15.7
d730	Relating with strangers	9	13.7
d449	Carrying, moving and handling objects, other specified and unspecified	7	13.7
e499	Attitudes, unspecified	7	13.7
d820	School education	7	11.8
b265	Touch function	6	11.8
d845	Acquiring, keeping and terminating a job	6	11.8
d110	Watching	5	11.8
d799	Interpersonal interactions and relationships, unspecified	5	11.8
d855	Non-remunerative employment	19	9.8
d166	Reading	13	9.8
b164	Higher-level cognitive functions	7	9.8
b765	Involuntary movement functions	6	9.8
d879	Economic life, other specified and unspecified (economic charge for the family)	9	7.8
e330	People in positions of authority	6	7.8
b144	Memory functions	5	7.8
b210	Seeing functions	5	7.8
d170	Writing	5	7.8
d330	Speaking	5	7.8
e425	Individual attitudes of acquaintances, peers, colleagues, neighbours and community members	5	7.8
b110	Consciousness functions	4	7.8
b240	Sensations associated with hearing and vestibular function	4	7.8
d475	Driving	4	7.8
d489	Moving around using transportation, other specified and unspecified	4	7.8
e315	Extended family	4	7.8
s770	Additional musculoskeletal structures related to movement	9	5.9
d138	Acquiring information	6	5.9
d175	Solving problems	6	5.9
d930	Religion and spirituality	6	5.9
d470	Using transportation	5	5.9
d720	Complex interpersonal interactions	5	5.9
b114	Orientation functions	3	5.9
b410	Heart functions	3	5.9
b449	Functions of the respiratory system, other specified and unspecified	3	5.9
b510	Ingestion functions	3	5.9
d660	Assisting others	3	5.9
b450	Additional functions of the respiratory system	2	5.9
b156	Perceptual functions	3	3.9
b230	Hearing functions	3	3.9
d710	Basic interpersonal interactions	3	3.9
b235	Vestibular functions	2	3.9
d729	General interpersonal interactions, other specified and unspecified	2	3.9
d779	Particular interpersonal relationships, other specified and unspecified	2	3.9
b749	Muscle functions, other specified and unspecified (flexibility)	1	3.9
e398	Support and relationships, other specified	1	3.9
b139	Global mental functions, other specified and unspecified	3	2.0
d179	Applying knowledge, other specified and unspecified (disease prevention)	2	2.0
b117	Intellectual functions	1	2.0
b460	Sensations associated with cardiovascular and respiratory functions	1	2.0
b599	Functions of the digestive, metabolic and endocrine systems, unspecified	1	2.0
b830	Other functions of the skin	1	2.0
b840	Sensation related to the skin	1	2.0
e455	Individual attitudes of other professionals	1	2.0
e498	Attitudes, other specified (criticism)	1	2.0
e590	Labour and employment services, systems and policies	1	2.0
s120	Spinal cord and related structures	1	2.0

ICF: International Classification of Functioning, Disability and Health.
